# Structural and Functional Characterization of NadR from *Lactococcus lactis*

**DOI:** 10.3390/molecules25081940

**Published:** 2020-04-22

**Authors:** Artem Stetsenko, Rajkumar Singh, Michael Jaehme, Albert Guskov, Dirk Jan Slotboom

**Affiliations:** Groningen Biomolecular Science and Biotechnology Institute, University of Groningen, Nijenborgh 4, 9747 AG Groningen, The Netherlands; a.stetsenko@rug.nl (A.S.); rajkumar.singh@rug.nl (R.S.); michael.jaehme@googlemail.com (M.J.)

**Keywords:** nicotinamide riboside, vitamins, NAD, NMN, NadR

## Abstract

NadR is a bifunctional enzyme that converts nicotinamide riboside (NR) into nicotinamide mononucleotide (NMN), which is then converted into nicotinamide adenine dinucleotide (NAD). Although a crystal structure of the enzyme from the Gram-negative bacterium *Haemophilus influenzae* is known, structural understanding of its catalytic mechanism remains unclear. Here, we purified the NadR enzyme from *Lactococcus lactis* and established an assay to determine the combined activity of this bifunctional enzyme. The conversion of NR into NAD showed hyperbolic dependence on the NR concentration, but sigmoidal dependence on the ATP concentration. The apparent cooperativity for ATP may be explained because both reactions catalyzed by the bifunctional enzyme (phosphorylation of NR and adenylation of NMN) require ATP. The conversion of NMN into NAD followed simple Michaelis-Menten kinetics for NMN, but again with the sigmoidal dependence on the ATP concentration. In this case, the apparent cooperativity is unexpected since only a single ATP is used in the NMN adenylyltransferase catalyzed reaction. To determine the possible structural determinants of such cooperativity, we solved the crystal structure of NadR from *L. lactis* (NadR_Ll_). Co-crystallization with NAD, NR, NMN, ATP, and AMP-PNP revealed a ‘sink’ for adenine nucleotides in a location between two domains. This sink could be a regulatory site, or it may facilitate the channeling of substrates between the two domains.

## 1. Introduction

Nicotinamide riboside (NR, a form of Vitamin B3) is one of the common precursors for biosynthesis of nicotinamide adenine dinucleotide (NAD) [1,2]. In some prokaryotes, NR is taken up by PnuC membrane transporters via a facilitated-diffusion transport mechanism [3,4,5,6,7,8]. Recently a high-resolution crystal structure of PnuC from *Neisseria mucosa* with a bound NR molecule was reported [9,10]. The substrate specificity of the PnuC protein for NR has been confirmed for homologs from *Escherichia coli, Haemophilus influenzae, Haemophilus parainfluenzae, Salmonella typhimurium,* and *Neisseria mucosa* [5,6,10,11,12]. In some organisms, such as *Clostridium thermocellum* and *Nostoc punctiforme,* the *pnuC* gene clusters with *nadR* [1,2]. NadR converts NR into nicotinamide mononucleotide (NMN) and subsequently into NAD [13]. NadR was initially thought to have a transport function too, which was later found to be incorrect [14]. Instead, the conversion of NR into NMN and subsequently into NAD [14] by NadR leads to metabolic substrate trapping of NR in the cytoplasm as phosphorylation prevents the efflux via PnuC. NadR is present in many Gram-positive and Gram-negative bacteria [15], and in some organisms, the protein also senses the internal NAD pool in order to repress essential genes for NAD biosynthesis and to regulate transport via PnuC in response to cellular NAD levels [16,17,18,19]. In Enterobacteria, the gene encoding PnuC is located in an operon with the gene for the NAD biosynthesis protein NadA (quinolinate synthase), and the expression of this gene cluster is regulated by NadR. In this case the bifunctional NadR enzyme acts additionally as a transcription factor due to the presence of an *N*-terminal DNA binding motif [15]. 

A structure of NadR from *Haemophilus influenzae* (NadR_Hi_) has been determined and revealed that the protein consists of two distinct domains separated by a short flexible linker, probably necessary for the movement of domains to perform both enzymatic steps [20,21,22]. Each domain has a specific catalytic activity: the *N*-terminal domain possesses an NMN adenylyltransferase (EC 2.7.7.1) activity (NMNAT domain), and the *C*-terminal domain is a ribosylnicotinamide kinase (EC 2.7.1.22) (RNK domain) (Supplementary Figure S1). Structurally the NMNAT domain is similar to archaeal NMNAT, whereas the RNK domain is similar to yeast thymidylate kinase [22]. For the NadR gene of *E. coli*, it has been shown experimentally that the NMNAT domain is indeed responsible for the transfer of the AMP moiety from ATP to NMN [23]. A crystal structure of the *E. coli* NMNAT domain of NadR with NAD bound has been reported [24]. NadR from *S. typhimurium* has been shown to be a NAD dependent repressor for several genes [*nadB, nadA-pnuC,* and *pncB*] involved in *de novo* biosynthesis of NAD and niacin salvage [16,25,26,27,28,29]. Additionally, there are some suggestions that NadR interacts with the PnuC membrane protein during transport of NR molecule [30]; however, so far, there is no experimental evidence for such direct interaction. 

The NadR enzyme from the Gram-negative bacterium *H. influenzae* is biochemically and structurally well characterized. Here, we report the first structural and biochemical characterization of a NadR protein from a Gram-positive bacterium (*Lactococcus lactis)*, and compare it with the Gram-negative ortholog. 

## 2. Results

### 2.1. Multiple Sequence Alignment of NadR

**Figure 1:**
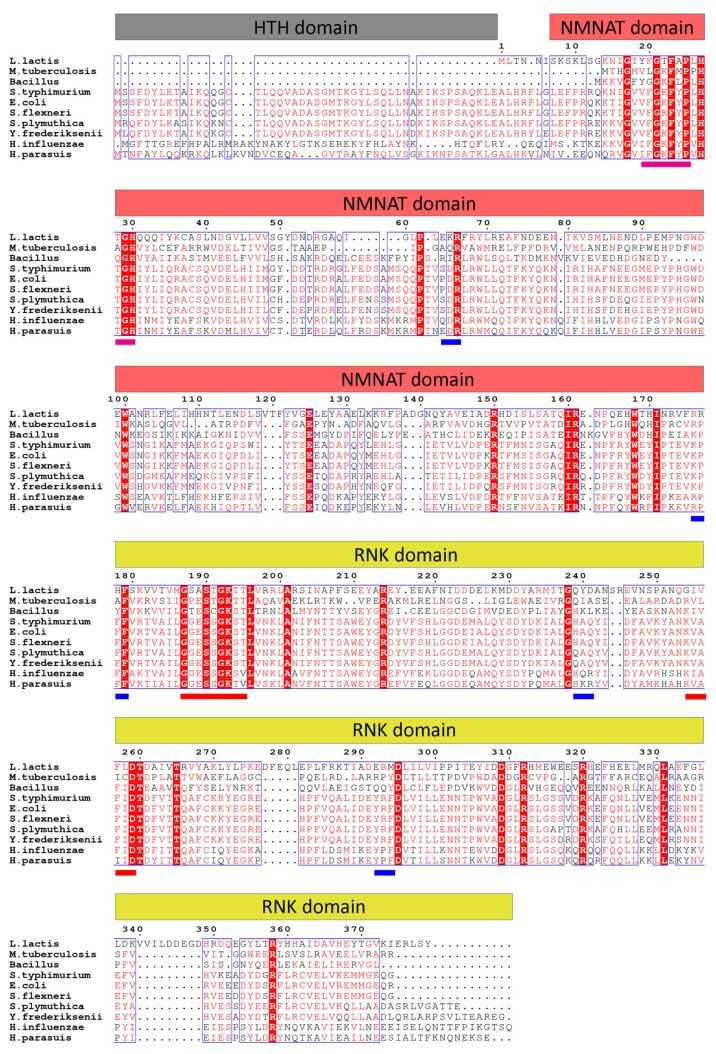
Multiple sequence alignment of NadR proteins from selected organisms. The amino acid residues are colored based on conservation, amino acids colored red are similar in more than 50% of the aligned NadR sequences. The amino acid residues in the red columns are conserved in all aligned NadR sequences. Organisms’ names: *L. lactis* (*Lactococcus lactis*), *M. tuberculosis* (*Mycobacterium tuberculosis*), *Bacillus* sp. (*Bacillus sp.*), *S. typhimurium* (*Salmonella typhimurium*), *E. coli* (*Escherichia coli*), S. *flexneri* (*Shigella flexneri*), S. *plymutica* (*Serretia plymutica*), Y. *frederiksenii* (*Yersenia frederiksenii*), *H. influenzae* (*Haemophilus influenzae*), and *H. parassuis* (*Haemophilus parassuis*). The bars on top show the locations of the three distinct domains of NadR proteins. The blue bars below the alignment indicate NAD binding residues, the red bars walker A and walker B motifs, and the pink bars are the phosphate-binding sites.

To compare the NadR_Ll_ protein sequence with the homologous proteins from different organisms, we did a multiple sequence alignment [31]. In contrast to many homologs, NadR_Ll_ lacks the *N*-terminal DNA binding domain (Figure 1, corresponding to amino acid residues 1 to 59 in the *H. influenza* sequence, also known as HTH domain responsible for DNA binding). NadR protein sequences are more conserved among Gram-negative bacteria (54.7% sequence identity between NadR_Ec_ (NadR protein from *E. coli*) and NadR_St_ (NadR protein from *S.typhimurium*)) compared to NadR proteins from Gram-positive bacteria (26.4% sequence identity between NadR_Ll_ and NadR_Bs_ (NadR protein *Bacillus* sp.). The sequence identity between NadR_Ec_ and NadR_Ll_ is about 23.5%.

### 2.2. Substrate Specificity of NadR

We performed isothermal titration calorimetry (ITC) measurements to study substrate binding to NadR_Ll_. We tested the binding of NR, NMN, NAD, ATP, and AMP-PNP (adenylyl-imidodiphosphate, an extremely slowly-hydrolyzable ATP analog). The measured binding affinities for Mg^2+^-complexed ATP and AMP-PNP are 63 µM and 13 µM, respectively (Table 1). Surprisingly, no binding for the nicotinamide nucleotide substrates in the presence and absence of ATP and AMP-PNP was detected. One possible explanation is that these substrates were bound very tightly to the protein during protein production in *E. coli*, and stayed attached during the protein purification steps. To circumvent this potential problem, we tried different unfolding-refolding protocols (including the use of urea and guanidium chloride up to a four molar concentration) of the purified protein in the absence of any substrate. However, upon treatment with denaturants, the protein stability decreased, and the protein could not be refolded (inferred from subsequent size exclusion chromatography). We also tried to perform mass-spectrometry on the purified protein in solution and on NadR_Ll_ protein crystals to determine whether there were tightly bound nucleotides; however, the results were inconclusive (although in the protein crystals we detected some traces of NR and NMN). 

### 2.3. Steady State Kinetics

To investigate the kinetics of the NadR_Ll_-catalyzed reaction, we used a lactate dehydrogenase (LDH) coupled assay. LDH was used to reduce the product NAD of the NadR_Ll_-catalyzed reaction to NADH, the concentration of which can be measured by the absorption at 340 nm. In these measurements, we ensured that the LDH-catalyzed reaction was not rate-limiting. The NadR_Ll_ activity was determined in the presence of different concentrations of NR, NMN, and Mg^2+^-complexed ATP. The Michaelis-Menten equation combined with a substrate inhibition model, or the Hill model [32] was fitted to the data.

**Figure 2:**
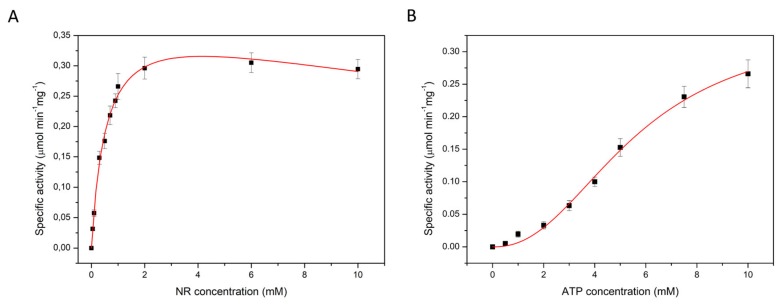
Apparent K_m_ and V_max_ determination of NadR from *L. lactis* for the substrate nicotinamide riboside at the constant ATP concentration of 10 mM (**A**) and for ATP at the constant NR concentration of 1 mM (**B**).

**Figure 3:**
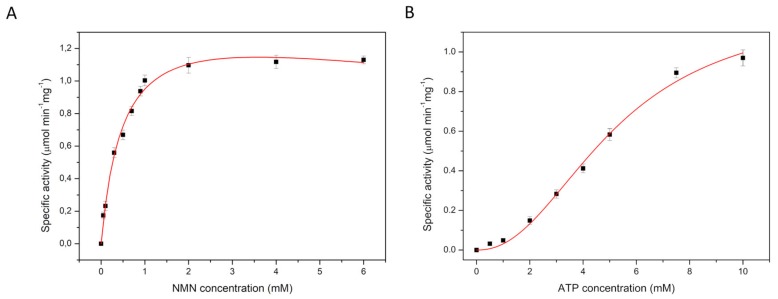
Apparent K_m_ and V_max_ determination for substrates nicotinamide mononucleotide (at a constant ATP concentration of 10 mM) (**A**) and ATP (at a constant NMN concentration of 1 mM) (**B**).

In measurements at constant ATP concentration (10 mM) the conversion of NR to NAD showed hyperbolic dependence on the NR concentration, but substrate inhibition was observed at high NR concentration. Analysis of the data using the Michaelis-Menten equation combined with a substrate inhibition model yielded K_m_ = 0.6 ± 0.1 mM, K_i_ = 31.4 ± 8.1 mM and V_max_ = 0.4 ± 0.1 µmol·min^−1^·mg^−1^ (Figure 2A and Table 2). The same experiment was performed with a fixed NR concentration of 1 mM, while varying the concentration of Mg-ATP. The Hill equation was fitted to the data, yielding K_m_ = 5.2 ± 0.9 mM for ATP and V_max_ of 0.4 ± 0.1 µmol·min^−1^·mg^−1^ with a Hill coefficient of 2.3 ± 0.5 (Figure 2B and Table 3). The value of the Hill coefficient implies that more than one molecule of ATP is cooperatively involved in NR conversion by NadR_Ll_ protein. The apparent cooperativity could be caused by the two-step catalysis, in which ATP is used for conversion of both NR to NMN, and NMN to NAD. To test this possibility, we also performed kinetic measurements using NMN as the substrate, and thus only following the second step, the conversion of NMN to NAD. At constant ATP concentration (10 mM) and varying concentration of NMN (up to 5 mM), the conversion of NMN to NAD followed Michaelis-Menten kinetics with substrate inhibition, with K_m_ = 0.5 ± 0.1 mM, K_i_ = 25.4 ± 10.8 mM and Vmax of 1.5 ± 0.1 µmol·min^−1^·mg^−1^ for NMN (Figure 3A and Table 2). Previously a lower K_m_ value for NMN conversion by the NadR_Hi_ protein was reported (0.14 mM) [21]. The discrepancy could be because the concentration of NMN used previously was varied only up to 1 mM with 0.2 mM of ATP present during that experiment. It could be that in these experimental conditions, the amount of ATP was limiting, but since no K_m_ or V_max_ values for ATP were reported, it is not possible to conclusively attribute the discrepancy to the ATP concentration. The differences could also simply reflect the unequal activity of NadR proteins from different organisms. The same experiment was performed using a fixed NMN concentration of 1 mM while varying the concentration of Mg-ATP up to 10 mM. The Hill equation was fitted to the data-yielding K_m_ of 5.2 ± 0.6 mM, for ATP with V_max_ of 1.2 ± 0.1 µmol·min^−1^·mg^−1^ and Hill coefficient of 2.2 ± 0.3 (Figure 3B and Table 3). Again, the Hill coefficient is an indication that more than one molecule of ATP is utilized during NMN conversion, which is surprising because only a single ATP molecule is used in the reaction from NMN to NAD. 

### 2.4. Dependence of the Kinetics of NAD Production on the NadR_ll_ Concentration

**Figure 4:**
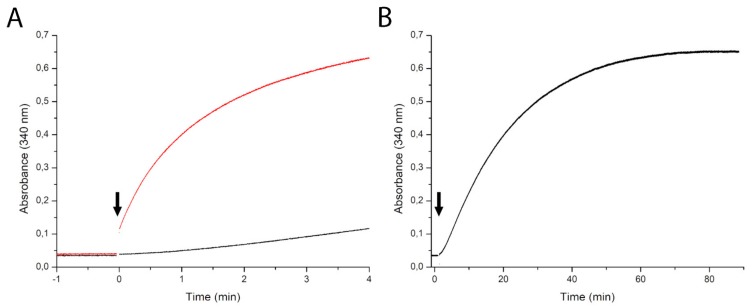
Time course of NADH formation with nicotinamide riboside as a substrate at two different protein concentrations (0.1 mg·mL^−1^ (black line) and 1.0 mg·mL^−1^ (red line), arrows indicate the addition of NadR) (**A**). Extended time scale (90 min) of the experiment with 0.1 mg·mL^−1^ (black line) (**B**).

To investigate further the reactions catalyzed by NadR_Ll_, we measured kinetics with 1 mM NR as a substrate using two different concentrations of protein (0.1 mg·mL^−1^ and 1.0 mg·mL^−1^). An increase in absorbance at 340 nm from NADH production was measured during the time course of the reaction. With the lower protein concentration (0.1 mg·mL^−1^) we observed lag phase, which can be explained because the NadR protein first converts NR to NMN as an intermediate product, which accumulates to some extent; subsequently, the conversion of NMN to NAD takes place that results in an increase in absorbance. With the ten-fold higher protein concentration (1.0 mg·mL^−1^), we observed that reaction goes faster (Figure 4A), and there is no visible build-up phase meaning that the rate of NMN production is not limiting the second step. When the reaction with 0.1 mg·mL^−1^ protein was allowed to run longer (more than 1 h), gradually, the amount of product reached almost the same level (Figure 4B). During this experiment, the LDH activity was not rate-limiting (data not shown). 

### 2.5. Oligomeric State of NadR

**Figure 5:**
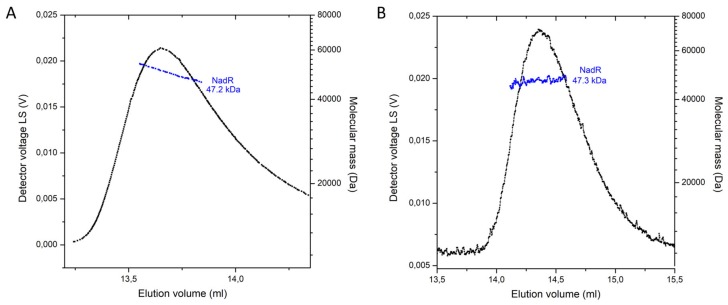
Oligomeric state of the NadR protein from *L. lactis* in solution. SEC-MALLS analysis of NadR without substrates (**A**) and in the presence of 5 mM ATP and 1 mM NMN (**B**).

Singh et al. [22], speculated that NadR_Hi_ may presumably work as a tetramer based on the crystal packing they observed in the structure of NadR_Hi_. This idea was corroborated by analytical ultracentrifugation experiments; however, the results showed some heterogeneity in the mass of complex [22]. A tetrameric arrangement creates a closed barrel with the cavity at the center and with the active sites of both domains (per monomer) facing away from the cavity [22]. To check the oligomeric state of NadR_Ll_, we used Multiangle laser light scattering coupled to Size-exclusion chromatography (SEC-MALLS), as this technique has been successfully used to determine oligomeric states of proteins [33,34]. SEC-MALLS showed that NadR_Ll_ with and without substrates is a monomeric protein with an average mass of 47.3 kDa in solution (Figure 5A,B). Notably, the presence of the substrate affected the elution volumes. A possible explanation can be that protein without substrate is conformationally more flexible, while binding to substrate makes it more globular, which yields a delayed elution volume.

### 2.6. Crystal Structures of NadR

**Figure 6:**
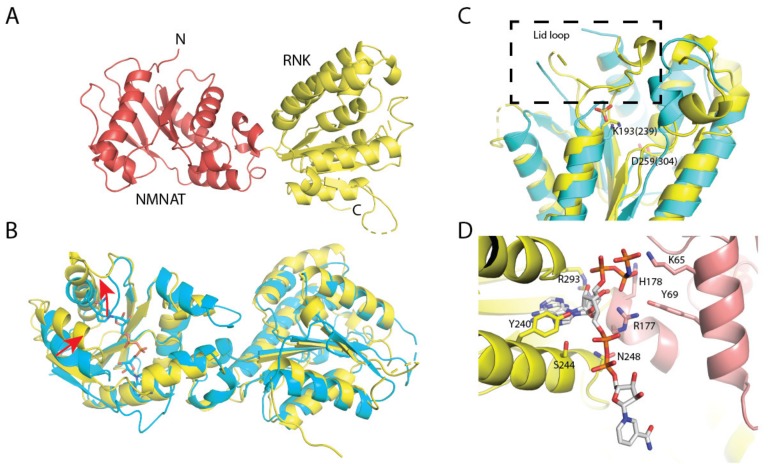
Crystal structure of NadR_Ll_. (**A**) Overall fold in secondary structure cartoon representation, NMN adenylyltransferase (NMNAT) domain in red and ribosylnicotinamide kinase (RNK) domain in yellow; unresolved loops are shown as dashed lines. (**B**) Superposition of NadR_Ll_ with NadR from *H. influenzae* (cyan, the substrate from this structure is shown as sticks), the movements of the nicotinamide-recognition loop and one of the helixes forming the binding site are shown with red arrows; rmsd ~ 2.5 Å; (**C**) ATP-binding site in the RNK domain: the position of the lid loop is indicated within a dashed rectangle; a sulfate ion likely occupies the position of one of ATP phosphates; essentially conserved Lys and Asp residues of Walker A and B motifs are shown as sticks (numbering from NadR_Ll_, with NadR_HI_ numbers in brackets); (**D**) A possible binding site at the interface between the domains: NAD, AMP-PNP, and the protein residues forming this binding site are shown as sticks. The omit electron densities for these cofactors, as well as NR and NMN, are shown in Supplementary Figure S2.

NadR_Ll_ crystallized in the P6_4_22 space group with two molecules in the asymmetric unit. The structure was solved using the structure of NadR_Hi_ (PDB code: 1LW7) as an input for the molecular replacement. After several rounds of refinement intertwined with manual model building, the final NadR_Ll_ model was obtained (see Table 4 for the detailed statistics on data collection and refinement). Each molecule of NadR_Ll_ is comprised of two domains—an *N*-terminal NMNAT domain (residues 8–177) folded in a Rossmann-like fold and a *C*-terminal RNK domain (residues 178–379) (Figure 6). In the previously reported NadR_Hi_ crystal structure, NAD was bound to the NMNAT domain; however, we did not observe NAD molecules bound to the NMNAT domain of NadR_Ll_. The clamshell-like binding site was rather squeezed, and the nicotinamide recognition loop was open (Figure 6B). The ATP-binding site of the NMNAT domain was also distorted (Figure 6C), and we observed a bound sulfate ion at the position where the β- or γ-phosphate of ATP is expected to be located. The RNK domain also had no substrate bound, with the positions of essentially conserved side chains of Walker A (residues 187–195) and Walker B (residues 254–260) motifs (canonical motifs for ATP-binding) of NadR_Ll_ and NadR_Hi_ superimposing well (Figure 6C, also see Figure 1 for the sequence alignment). The lid region (residues 304–318) had fallen back into the binding site (Figure 6C), thus, there is not enough space for binding of ATP or NR.

Interestingly, we observed strong positive electron density in the region between the two domains whenever we added substrates for co-crystallization. In one of the collected datasets, we observed a mixture of NAD and AMP-PNP bound at this location in the electron density (Figure 6D and Supplementary Figure S2). Additionally, we obtained structures in the presence of NMN and NR, which both have bound substrates in a similar area. This novel ‘binding site’ is very different from the canonical binding sites in NMNAT and RNK domains and is formed by side chains of K65, Y69, R177, H178, Y240, S244, N248, and R293 (Figure 6D). 

## 3. Discussion

NadR proteins can be involved in different functions: NAD biosynthesis and transcriptional regulation. While NadR proteins from *Haemophilus* sp. and most Gram-negative bacteria contain a DNA binding domain at the N terminus, such a domain is absent in the NadR_Ll_ protein, indicating that NadR_Ll_ is not involved in transcriptional regulation. Here we provide a biochemical study of NadR_Ll_ along with crystal structures. We found sigmoidal dependence of the rate of NAD formation on the concentration of ATP, both for the conversion of NR to NAD and for the conversion of NMN to NAD. Whereas the sigmoidal behavior could be explained for the reaction in which NR is converted into NAD, because two molecules of ATP are used, the sigmoidal behavior is unexpected for the conversion of NMN. There is possibly a regulatory site where ATP binds. The positive electron density between the two domains observed in the crystal structure could be such a site. 

We solved crystal structures of NadR_Ll_ in the presence of various substrates. The only available crystal structure of NadR_Hi_ contains bound NAD in both the domains. Here we observed electron density for an NAD or other nucleotide molecule at a different site, located between the two domains, whereas the predicted active sites were unoccupied. Although we cannot completely rule out that the new binding site is an artifact of crystallization, another explanation is that this site is a transfer hub between two domains. After the RNK domain has performed phosphorylation of nicotinamide riboside to produce NMN, the latter molecule has to be transported to the NMNAT domain, where the consequent conversion into NAD takes place. Such a depot site might be of use to temporarily keep an intermediate substrate to prevent its diffusion away from the protein. The site may also have a function for allosteric regulation, which could explain the sigmoidal dependence of the conversion of NMN to NAD on the ATP concentration.

## 4. Materials and Methods

### 4.1. Cloning

The gene encoding NadR_Ll_ was cloned in the p2BAD vector [35] using the restriction sites *Sac*1 and *Kas*1 with the sequence coding for an *N*-terminal twin strep tag (WSHPQFEK-(GGGS)2-GGS-SA-WSHPQFEK) downstream of the second promotor. The plasmid was transformed into competent cells of the *E. coli* strain MC1061.

### 4.2. Protein Expression and Purification 

Protein overexpression was done in the *E. coli* MC1061 strain transformed with the expression plasmid. Cells were cultivated in five-liter flasks containing 2 L luria bertani (LB) medium with the composition of 10 g NaCl, 10 g Tryptone, and 5 g yeast extract per liter. The *E. coli* cells with p2BAD plasmid were grown at 37 °C, 200 rpm to an OD600 of 0.6. Once the OD reached 0.6, induction was done with 0.04% arabinose. After induction, cells were grown further for another 3 h at 37 °C. After 3 h of induction, cells were collected by centrifugation (20 min, 7,446 g, 4 °C), washed in wash buffer (50 mM Tris/HCl, pH 7.5) and resuspended in the buffer A (50 mM Tris/HCl, pH 7.5, 150 mM NaCl, and 10% glycerol). Cells were lysed by high-pressure disruption (Constant Cell Disruption System Ltd., Northamptonshire UK, one passage at 25 kPsi for *E. coli* cells at 5 °C). After cell lysis, 1 mM MgSO4, 0.2 mM PMSF, and 50–100 mg·mL^−1^ DNase were added. Cell debris was removed by low-speed centrifugation (20 min, 12,074 g, 4 °C). To remove crude membranes and other impurities, the supernatant was centrifuged for 45 min, 193727 g, 4 °C. The clear supernatant was used for further protein purification.

The clear supernatant was passed through a streptavidin resin column (total volume 1 mL), which was pre-equilibrated with buffer B (50 mM Tris/HCl, pH 8.0 and 1 mM EDTA). The flow-through was collected for SDS gel electrophoresis. Once supernatant was passed, the column material was washed with 20 CV of wash buffer C (50 mM Tris/HCl pH 8.0, 200 mM NaCl, 1 mM EDTA). NadR was eluted in three fractions (E1, E2 and E3) of 350, 750 and 600 µL respectively with elution buffer D (50 mM Tris/HCl pH 8.0, 150 mM NaCl, 1 mM EDTA and 1 mM des-thiobiotine). Elution fraction E2 contained the highest amount of protein as measured by absorbance at 280 nm using a nanodrop machine. This fraction was further purified by size-exclusion chromatography with column Superdex 200, 10/300 gel filtration column (GE Healthcare), equilibrated with buffer E (50 mM Tris/HCl pH 8.0, 150 mM NaCl).

After size-exclusion chromatography, the fractions containing the NadR protein were combined and used directly for ITC and kinetics measurements. For crystallization, the protein was concentrated with a vivaspin 500 concentrator with a molecular weight cutoff of 30 kDa (Sartorius stedim), to a final concentration of 17 mg·mL^−1^.

### 4.3. Isothermal Calorimetric Titration (ITC) Measurements for Substrate Specificity

ITC measurements were conducted with an ITC200 calorimeter (MicroCal, Malvern, UK) at 25 °C. The substrates nicotinamide riboside (Niagen TM, Chromadex, Los Angeles, CA, USA), NMN (Sigma Aldrich, St. Louis, MO, USA), NAD (Sigma Aldrich, St. Louis, MO, USA) and nicotinamide were dissolved in the buffer that was used for protein purification. The substrate concentration used for ITC measurements varied from 1 mM to 5 mM as a final concentration. The ligand solutions were added into the temperature-equilibrated ITC cell filled with ~300 μL of protein with a concentration of 10–50 µM. The final obtained data were analyzed with ORIGIN-based software (MicroCal).

### 4.4. Lactate Dehydrogenase (LDH) Coupled NADH Measurements Assay 

NadR_Ll_ kinetics was assayed using lactate dehydrogenase (LDH) coupled NADH measurements using an Agilent spectrophotometer (Cary 100 UV-Vis) with a quartz cuvette. The measurements were done by varying the concentration of NR, in the reaction buffer F (100 mM Tris/HCl pH 7.5) containing 10 mM Mg-ATP, 150 mM lactic acid, 10 µL (10 mg·mL^−1^, 550 units/mg) of LDH and NadR_Ll_ protein with a concentration of 1.0 mg·mL^−1^, with a final volume of 200 µL. The same reaction was also performed, by varying the concentration of Mg-ATP (up to 10 mM) while keeping NR concentration constant at 1 mM in the presence of 150 mM lactic acid, 10 µL (10 mg·mL^−1^, 550 units/mg) of LDH and 1.0 mg·mL^−1^ of NadR_Ll_ protein in the buffer F with the final volume of 200 µL. For NMN measurements, we varied the concentration up to 5 mM, and we used 10 mM Mg-ATP, 150 mM lactic acid, 10 µL (10 mg·mL^−1^, 550 units/mg) of LDH, and 1.0 mg·mL^−1^ NadR_Ll_ protein in the buffer F with the same reaction volume of 200 µL. The reaction was further studied with the fixed amount of NMN (1 mM) and varying Mg-ATP concentrations, as described for NR. The reaction was started by the addition of NadR protein to all other pre-mixed components (incubated at 25 °C for 1 min), and NADH absorbance was measured at a wavelength of 340 nm. As controls, each component was removed one by one (with the appropriate volume compensation by addition of buffer) during the kinetics measurements.

The specific enzyme activity was calculated by using the formula, as shown below

(1) $$A_{sp}=\frac{\delta \times V_{rxn}\times 1000}{6220\times V_{enz\;}\times c}$$

where: $A_{sp}$ is initial specific activity (µmol·min^−1^·mg^−1^); δ is the initial slope of increase in absorbance (min^−1^); $V_{rxn}$ is total reaction volume (µL); $V_{enz}$ is added diluted enzyme volume (µL); c is protein stock concentration (mg·mL^−1^).

$V_{max}$ and $K_{m}$ values were determined using either classical or modified (when the substrate inhibition was observed) Michaelis-Menten equations

(2) $$V_{0}=\frac{V_{max}\times S}{K_{m}+S}or\;V_{0}=\frac{V_{max}\times S}{K_{m}+S+\frac{S^{2}}{K_{i}}}$$

where: $V_{0}$ is initial specific activity measured in initial step of reaction (µmol·min^−1^·mg^−1^); $V_{max}$ is maximal possible specific rate of reaction (µmol·min^−1^·mg^−1^); S is substrate concentration (mM); $K_{m}$ is michaelis-Menten constant (mM); $K_{i}$ is substrate inhibition concentration (mM).

The hill fitting parameters were calculated using the following equation

(3) $$V_{0}=\frac{V_{max}\times S^{n}}{K_{m}{}^{n}+S^{n}}$$

where n is the hill coefficient.

Origin Pro v8.0724 Software (OriginLab Corporation) was used for the non-linear fitting of data according to this equation.

### 4.5. Crystallization

The initial crystallization trials were done with the commercial screens such as MCSG (Microlytic, Burlington, MA, USA) and Membrane Gold (Molecular Dimensions, Sheffield, UK) using the mosquito crystallization robot (TPP LabTech, Melbourne, UK). Initial crystals were obtained at 4 °C, but the diffraction was limited to 7–10 Å resolution. After the first round of optimization, we improved the diffraction up to 5 Å resolution. Further optimizations were set up as vapor-diffusion sitting drops (1:1 ratio, 0.1 µL) at 16 °C with the protein concentration of 17 mg·mL^−1^. Diamond-shaped crystals were obtained within 3–5 days. These crystals diffracted to around 3 Å resolution. As the final optimization, the additive screen (Hampton Research) was used to obtain the crystals with the diffraction limit of 2–3 Å resolution. The crystallization conditions were as follows: 1 M (NH_4_)_2_SO_4_, 100 mM Na Citrate pH 6.5, 10 mM MgCl_2_ and 10% glucose or 1 M (NH_4_)_2_SO_4_, 100 mM Hepes pH 7.0 and 10% CaCl_2_. Co-crystallization with NR, NMN, NAD, Mg-ATP, and with Mg-AMP-PNP was performed. In the NR-bound state, the co-crystallization condition was 1 M (NH_4_)_2_SO_4_, 100 mM Na Citrate pH 6.5, 5 mM NR, 10 mM MgCl_2,_ and 10% glucose. In NMN bound co-crystallization condition, all the components mentioned above remained the same except NR was replaced by the addition of 5 mM NMN. The NAD bound co-crystallization condition also remained the same with an addition of 5 mM NAD along with 5 mM Mg-AMP-PNP. All the crystals were flash-frozen in liquid nitrogen for further X-ray diffraction analysis.

### 4.6. Static Light Scattering and Refractive Index Measurements 

The determination of the oligomeric state of purified NadR_Ll_ was performed as described [33,34]. Briefly, the Superdex 200 column used for the SEC-MALLS analysis was equilibrated with SEC buffers (50 mM Tris/HCl, pH 8.0, 150 mM NaCl or 50 mM Tris/HCl, pH 8.0, 150 mM NaCl, 5 mM ATP, 1 mM NMN, filtered through 0.1 μm pore size VVLP filters (Millipore)), and subsequently the buffer was recirculated through the system for 16 h at 0.5 mL/min. Purging of the refractometer was switched off when the baselines were stable, and the detector reading was set to zero. 200 µL of protein solutions (0.5 mg·mL^−1^) with and without substrates accordingly were injected, and the data from the three detectors were imported by the ASTRA software package (Wyatt Technologies, Santa Barbara, CA, USA). The experiments were done at room temperature, but the differential refractometer was precisely set at 30 °C.

### 4.7. Data Collection, Structure Determination, and Refinement 

The diffraction data were collected at 100 K at the PX beamline at SLS (Villigen, Switzerland) and at the beamline ID29 (ESRF, Grenoble, France). Collected data were processed with XDS package [36]. Molecular replacement was performed with Phaser [37] using NadR from *H. influenzae* (PDB code 1LW7) as a starting model. Refinement was performed in Phenix [38] with the manual corrections in Coot [39]. The composite omit maps were calculated using simulated annealing protocol implemented in Phenix [38]. The final models were deposited in PDB databank under 6GYE for NadR with NR, 6GYF for NadR with NMN, and 6GZO for NadR with NAD/AMP-PNP.

## Figures and Tables

**Table 1:** Substrate binding studied by isothermal titration calorimetry (ITC) with NadR protein from *L. lactis*.

Substrate	Dissociation Constant–K_D_ (µM)
ATP	63 ± 1
AMP-PNP	13 ± 1
NR	No Binding
NMN	No Binding
NAD	No Binding
Nicotinamide	No Binding
Nicotinic acid	No Binding

The error represents the upper and lower values from two measurements.

**Table 2:** Apparent K_m,_ K_i_ and V_max_ values of the NadR-catalyzed reaction for nicotinamide mononucleotide (NMN) and nicotinamide riboside (NR) in presents of constant concentration ATP (10 mM).

	K_m_, mM	V_max_, µmol·min^−1^·mg^−1^	K_i_, mM
NR	0.6 ± 0.1	0.4 ± 0.1	31.4 ± 8.1
NMN	0.5 ± 0.1	1.5 ± 0.1	25.4 ± 10.8

The error is the standard derivations of the three independent measurements.

**Table 3:** Apparent K_m_, V_max_ and the Hill coefficient values of NadR from *L. lactis* for ATP in the presence of a constant concentration of NR or NMN (1mM ).

	Km, mM	Vmax, µmol·min^−1^·mg^−1^	The Hill Coefficient
NR	5.2 ± 0.9	0.4 ± 0.1	2.3 ± 0.5
NMN	5.2 ± 0.6	1.2 ± 0.1	2.2 ± 0.3

The error is the standard derivations of the three independent measurements.

**Table 4:** Data collection and refinement statistics for the NadR_Ll_ crystal structures with respective substrates.

	NadR NR	NadR NMN	NadR NAD AMP-PNP
PDB code Data collection	6GYE	6GYF	6GZO
Space group	P 6_4_ 2 2	P 6_4_ 2 2	P 6_4_ 2 2
Unit cell dimensions			
*a, b, c* (Å)	165.14, 165.14, 196.55	165.09, 165.09, 193.05	167.32, 167.32, 192.81
*α, β, γ* (°)	90.0, 90.0, 120.0	90.0, 90.0, 120.0	90.0, 90.0, 120.0
Resolution range (Å)	47.4–2.30 (2.33–2.30) *	47.83–2.70 (2.76–2.70) *	45.74–3.00 (3.09–3.00) *
*R*_meas_ (%)	16.6 (>100) *	13.1 (>100) *	9.4 (>100) *
*cc_1/2_*	99.8 (29.4) *	99.8 (33.0) *	99.8 (72.5) *
*I*/*σI*	12.5 (0.9) *	11 (0.6) *	5.6 (1.7) *
Completeness (%)	100 (100) *	100 (99.9) *	100 (99.4) *
Multipilicity	13.5 (13.4) *	13.4 (13.1) *	6.5 (6.4) *
Refinement			
Resolution (Å)	47.4–2.30	47.83–2.70	45.74–3.00
No. of reflections	70441	41577	32439
*R*_work_/*R*_free_	0.18/0.21	0.20/0.24	0.19/0.24
Number non-hydrogen atoms	6446	6109	5993
Protein	6084	6042	5838
Ligands/Ions	36/60	44/23	88/5
Water	266	0	0
*B*-factors (Å^2^)			
Protein	70.9	86.5	118.8
Ligand/Ions	109.7/115.1	98.6/101.1	135.9/178.3
Water	67.1	–	–
R.m.s. deviations			
Bond lengths (Å)	0.008	0.009	0.009
Bond angles (°)	0.958	1.054	1.275
Molprobity score	1.83	2.08	2.56

* for the highest resolution shell.

## Supplementary Materials

The following are available online, Figure S1: Schematic representation of NadR catalytic reaction, Figure S2: The composite omit electron density for (**A**) NR (contoured at 1σ) (**B**) NMN (contoured at 1.5σ) and (**C**) mixture of NAD and AMP-PNP (contoured at 1σ).

## References

[1] Jaehme M., Slotboom D.J. (2015). Structure, function, evolution, and application of bacterial Pnu-type vitamin transporters. Biol. Chem..

[2] Jaehme M., Slotboom D.J. (2015). Diversity of membrane transport proteins for vitamins in bacteria and archaea. Biochim. Biophys. Acta (BBA)-Gene. Subj..

[3] Bacher A., Eberhardt S., Fischer M., Kis K., Richter G. (2000). Biosynthesis of vitamin b2 (riboflavin). Annu. Rev. Nutr..

[4] Marsili E., Baron D.B., Shikhare I.D., Coursolle D., Gralnick J.A., Bond D.R. (2008). Shewanella secretes flavins that mediate extracellular electron transfer. Proc. Natl. Acad. Sci. USA.

[5] Kemmer G., Reilly T.J., Schmidt-Brauns J., Zlotnik G.W., Green B.A., Fiske M.J., Herbert M., Kraiss A., Schlor S., Smith A. (2001). NadN and e (P4) are essential for utilization of NAD and nicotinamide mononucleotide but not nicotinamide riboside in Haemophilus influenzae. J. Bacteriol..

[6] Grose J.H., Bergthorsson U., Xu Y., Sterneckert J., Khodaverdian B., Roth J.R. (2005). Assimilation of nicotinamide mononucleotide requires periplasmic AphA phosphatase in Salmonella enterica. J. Bacteriol..

[7] Rodionov D.A., Li X., Rodionova I.A., Yang C., Sorci L., Dervyn E., Martynowski D., Zhang H., Gelfand M.S., Osterman A.L. (2008). Transcriptional regulation of NAD metabolism in bacteria: Genomic reconstruction of NiaR (YrxA) regulon. Nucleic Acids Res..

[8] Jaehme M., Singh R., Garaeva A.A., Duurkens R.H., Slotboom D.-J. (2018). PnuT uses a facilitated diffusion mechanism for thiamine uptake. J. Gene. Physiol..

[9] Jaehme M., Guskov A., Slotboom D.J. (2014). Crystal structure of the vitamin B3 transporter PnuC, a full-length SWEET homolog. Nat. Struct. Mol. Biol..

[10] Slotboom D.J. (2014). Structural and mechanistic insights into prokaryotic energy-coupling factor transporters. Nat. Rev. Microbiol..

[11] Sauer E., Merdanovic M., Mortimer A.P., Bringmann G., Reidl J. (2004). PnuC and the utilization of the nicotinamide riboside analog 3-aminopyridine in Haemophilus influenzae. Antimicrob. Agents Chemother.

[12] Cynamon M.H., Sorg T.B., Patapow A. (1988). Utilization and Metabolism of NAD by Haemophilus parainfluenzae. Microbiology.

[13] Merdanovic M., Sauer E., Reidl J. (2005). Coupling of NAD+ biosynthesis and nicotinamide ribosyl transport: Characterization of NadR ribonucleotide kinase mutants of Haemophilus influenzae. J. Bacteriol..

[14] Grose J.H., Bergthorsson U., Roth J.R. (2005). Regulation of NAD synthesis by the trifunctional NadR protein of Salmonella enterica. J. Bacteriol..

[15] Gerasimova A.V., Gelfand M.S. (2005). Evolution of the NadR regulon in Enterobacteriaceae. J. Bioinform. Comput. Biol..

[16] Spector M.P., Hill J.M., Holley E.A., Foster J.W. (1985). Genetic characterization of pyridine nucleotide uptake mutants of Salmonella typhimurium. J. Gen. Microbiol..

[17] Tirgari S., Spector M.P., Foster J.W. (1986). Genetics of NAD metabolism in Salmonella typhimurium and cloning of the nadA and pnuC loci. J. Bacteriol..

[18] Zhu N., Roth J.R. (1991). The nadI region of Salmonella typhimurium encodes a bifunctional regulatory protein. J. Bacteriol..

[19] Vogl C., Grill S., Schilling O., Stulke J., Mack M., Stolz J. (2007). Characterization of riboflavin (vitamin B2) transport proteins from Bacillus subtilis and Corynebacterium glutamicum. J. Bacteriol..

[20] Mitchell P., Moyle J. (1958). Group-translocation: A consequence of enzyme-catalysed group-transfer. Nature.

[21] Kurnasov O.V., Polanuyer B.M., Ananta S., Sloutsky R., Tam A., Gerdes S.Y., Osterman A.L. (2002). Ribosylnicotinamide kinase domain of NadR protein: Identification and implications in NAD biosynthesis. J. Bacteriol..

[22] Singh S.K., Kurnasov O.V., Chen B., Robinson H., Grishin N.V., Osterman A.L., Zhang H. (2002). Crystal structure of Haemophilus influenzae NadR protein. A bifunctional enzyme endowed with NMN adenyltransferase and ribosylnicotinimide kinase activities. J. Biol. Chem..

[23] Raffaelli N., Lorenzi T., Mariani P.L., Emanuelli M., Amici A., Ruggieri S., Magni G. (1999). The *Escherichia coli* NadR regulator is endowed with nicotinamide mononucleotide adenylyltransferase activity. J. Bacteriol..

[24] Zhang H., Zhou T., Kurnasov O., Cheek S., Grishin N.V., Osterman A. (2002). Crystal structures of *E. coli* nicotinate mononucleotide adenylyltransferase and its complex with deamido-NAD. Structure (Lond. Engl. 1993).

[25] Penfound T., Foster J.W. (1999). NAD-dependent DNA-binding activity of the bifunctional NadR regulator of Salmonella typhimurium. J. Bacteriol..

[26] Foster J.W., Penfound T. (1993). The bifunctional NadR regulator of Salmonella typhimurium: Location of regions involved with DNA binding, nucleotide transport and intramolecular communication. FEMS Microbiol. Lett..

[27] Cookson B.T., Olivera B.M., Roth J.R. (1987). Genetic characterization and regulation of the nadB locus of Salmonella typhimurium. J. Bacteriol..

[28] Zhu N., Olivera B.M., Roth J.R. (1989). Genetic characterization of the pnuC gene, which encodes a component of the nicotinamide mononucleotide transport system in Salmonella typhimurium. J. Bacteriol..

[29] Foster J.W., Holley-Guthrie E.A., Warren F. (1987). Regulation of NAD metabolism in Salmonella typhimurium: Genetic analysis and cloning of the nadR repressor locus. Mol. Gen. Genet. (MGG).

[30] Zhu N., Olivera B.M., Roth J.R. (1991). Activity of the nicotinamide mononucleotide transport system is regulated in Salmonella typhimurium. J. Bacteriol..

[31] Robert X., Gouet O. (2014). Deciphering key features in protein structures with the new ENDscript server. Nucleic Acid Res..

[32] Hill A.V. (1910). The possible effects of the aggregation of the molecules of haemoglobin on its dissociation curves. J. Physiol (London).

[33] Slotboom D.J., Duurkens R.H., Olieman K., Erkens G.B. (2008). Static light scattering to characterize membrane proteins in detergent solution. Methods.

[34] Beek J.ter, Duurkens R.H., Erkens G.B., Slotboom D.J. (2011). Quaternary structure and functional unit of energy coupling factor (ECF)-type transporters. J. Biol. Chem..

[35] Birkner J.P., Poolman B., Koçer A. (2012). Hydrophobic gating of mechanosensitive channel of large conductance evidenced by single-subunit resolution. Proc. Natl. Acad. Sci. USA.

[36] Kabsch W. (2010). XDS. Acta Crystallogr. D.

[37] McCoy A.J., Grosse-Kunstleve R.W., Adams P.D., Winn M.D., Storoni L.C., Read R.J. (2007). Phaser crystallographic software. J. Appl. Crystallogr..

[38] Adams P.D., Afonine P.V., Bunkóczi G., Chen V.B., Davis I.W., Echols N., Headd J.J., Hung L.-W., Kapral G.J., Grosse-Kunstleve R.W. (2010). PHENIX: A comprehensive Python-based system for macromolecular structure solution. Acta Crystallogr. D.

[39] Emsley P., Lohkamp B., Scott W.G., Cowtan K. (2010). Features and development of Coot. Acta Crystallogr. D Biol. Crystallogr..

