# Supplementary material for: Structural and Functional Characterization of NadR from Lactococcus lactis
Source: Molecules. 2020 Apr 22;25(8):1940. doi: 10.3390/molecules25081940 (PMC7221760; doi:10.3390/molecules25081940)
Supplement: Supplementary file 1 [file molecules-25-01940-s001.pdf]

Structural and functional characterization of NadR from *Lactococcus lactis*

Artem Stetsenko, Rajkumar Singh, Michael Jaehme, Albert Guskov, Dirk Jan Slotboom

Supplementary Information

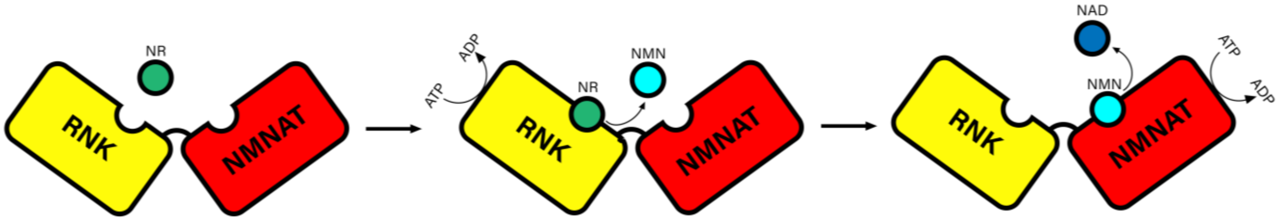

Supplementary Figure S1. Schematic representation of NadR catalytic reaction.

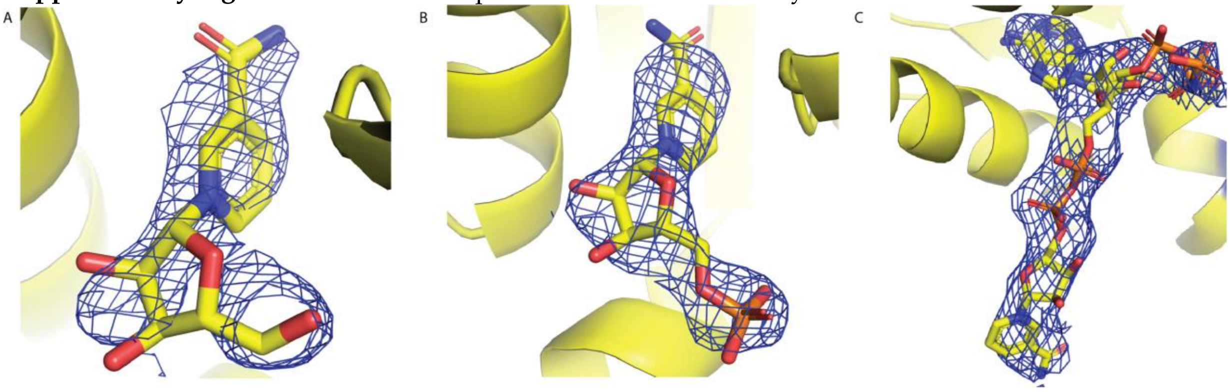

Supplementary Figure S2. The composite omit electron density for (**A**) NR (contoured at 1σ) (**B**) NMN (contoured at 1.5σ) and (**C**) mixture of NAD and AMP-PNP (contoured at 1σ)
